# Histology of “placoderm” dermal skeletons: Implications for the nature of the ancestral gnathostome

**DOI:** 10.1002/jmor.20119

**Published:** 2013-02-02

**Authors:** Sam Giles, Martin Rücklin, Philip C.J. Donoghue

**Affiliations:** ^1^ School of Earth Sciences, University of Bristol, Wills Memorial Building, Queen's Road, Bristol BS8 1RJ, UK; ^2^ Present address: Sam Giles is currently at Department of Earth Sciences, University of Oxford, Oxford, UK

**Keywords:** histology, placoderm, dermoskeleton, gnathostome, evolution, bone

## Abstract

The vertebrate dermal skeleton has long been interpreted to have evolved from a primitive condition exemplified by chondrichthyans. However, chondrichthyans and osteichthyans evolved from an ancestral gnathostome stem‐lineage in which the dermal skeleton was more extensively developed. To elucidate the histology and skeletal structure of the gnathostome crown‐ancestor we conducted a histological survey of the diversity of the dermal skeleton among the placoderms, a diverse clade or grade of early jawed vertebrates. The dermal skeleton of all placoderms is composed largely of a cancellar architecture of cellular dermal bone, surmounted by dermal tubercles in the most ancestral clades, including antiarchs. Acanthothoracids retain an ancestral condition for the dermal skeleton, and we record its secondary reduction in antiarchs. We also find that mechanisms for remodeling bone and facilitating different growth rates between adjoining plates are widespread throughout the placoderms. J. Morphol., 2013. © 2013 Wiley Periodicals, Inc.

## INTRODUCTION

**Figure 1:**
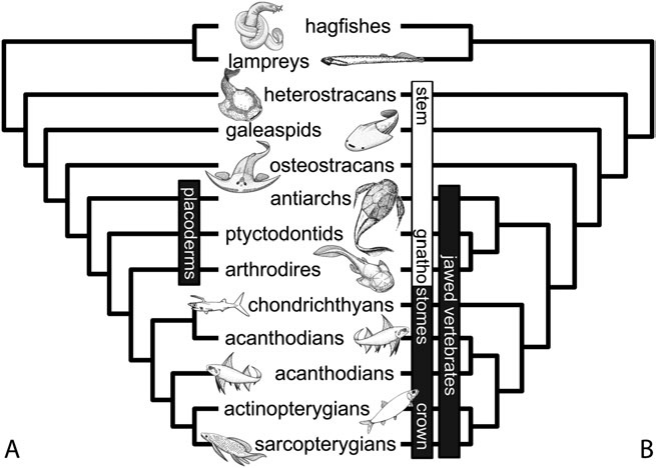
Evolutionary relationships of principal groups of vertebrates. Phylogenetic relationships among the principal groups of stem‐ and crown‐gnathostomes. The more recent hypothesis of placoderm paraphyly (**A**) vs. the traditional view of placoderm monophyly (**B**).

Among living vertebrates, only the gnathostomes (the clade of living jawed vertebrates) possess a mineralized skeleton. The gnathostome clade comprises chondrichthyans (sharks, rays, and holocephalans) and osteichthyans (all other living jawed vertebrates). Naturally, therefore, chondrichthyans have been pivotal in attempts to understand skeletal evolution among osteichthyans (Williamson, 1849, 1851; Hertwig, 1874a, b, 1876, 1879, 1882; Stensiö, 1961; Ørvig, 1951, 1968, 1977; Reif, 1976, 1978a, b, 1980, 1982). To be sure, chondrichthyans are a natural outgroup to osteichthyans, but they do not necessarily reflect an ancestral gnathostome state. This expectation is true of the chondrichthyan skeleton, and most especially of the micromeric dermal skeleton, which is reduced with respect to the primitive gnathostome condition, as evidenced by successive extinct sister lineages to the chondrichthyans, osteichthyans and, indeed, to crown gnathostomes (Donoghue and Sansom, 2002). These lineages include the jawed acanthodians and placoderms, and the jawless ostracoderms (osteostracans, galeaspids, heterostracans, thelodonts, and anaspids), all of which possessed an extensively developed mineralized dermal skeleton composed of bone and, in many lineages, dermal tubercles comprising enameloid and dentine (Donoghue and Sansom, 2002). With respect to the histological structure of the dermal skeleton in the gnathostome crown ancestor, the most poorly known and yet most significant of these extinct lineages are the placoderms, the evolutionary relationships of which remain the focus of vigorous debate (Fig. 1; e.g., Johanson 2002; Brazeau, 2009; Young, 2010; Davis et al., 2012). Perceived as a clade, Placodermi has been most widely considered a sister lineage to crown‐gnathostomes (Fig. 1A; Young, 2008, 2010). Perceived as an evolutionary grade, placoderms comprise a series of successive sister‐lineages to crown‐gnathostomes (Fig. 1B; Johanson, 2002; Brazeau, 2009; Davis et al., 2012). In either instance, placoderms remain integral to understanding the histological structure of the dermal skeleton in the ancestral crown gnathostome, the condition from which both chondrichthyans and osteichthyans are derived. Thus, we set out to survey the histology of the dermoskeleton across placoderm diversity. In providing an insight into the tissues present in the placoderms, we attempted to resolve the plesiomorphic condition of the gnathostome dermal skeleton and, in consequence, provide insight into the subsequent evolution of this skeletal system in extant vertebrates.

### Synthesis of Previous Research into the Histological Structure of the Placoderm Dermal Skeleton

Research into the microstructure of placoderm dermal armour has been ongoing since the 19th century (e.g., Agassiz, 1844; Gürich, 1891). However, there has been no systematic attempt to survey skeletal composition and reports are disparate both in their taxonomic and anatomical coverage, with little comprehensive understanding of how tissues and structures are distributed across different placoderm groups. Here, we review the sum of published knowledge.

#### Arthrodira

The dermal skeleton is perceived generally to consist of a superficial compact lamellar layer (referred to as the “Oberflächenschicht” by Heintz, 1929, or, within phyllolepids, as the “Skulpturschicht” by Gross, 1934), a cancellous spongiosa, and a compact lamellar base. The presence of distinct layers and their relative thickness varies from one account to another; in *Phyllolepis*, Stensiö (1934) notes that the superficial layer is confined to the ornament, and Hills (1931) identifies a multilayered basal layer. Heintz (1929) recognized two distinct layers of spongiosa within the dermal armour of *Heterogaspis* (*Monaspis*; contra Downs and Donoghue, 2009): a superficial‐most “Maschenschicht” with irregular interconnected cavities, and a basal‐most “Kanalschicht” with cavities typically parallel to the basal surface. The spongiosa is also purported to contain Haversian canals (Bystrow, 1957). Superficial tubercles are widespread in arthrodires. These may be composed of bone or semidentine, the latter of which is a putative synapomorphy of placoderms (Goujet and Young, 2004; Young, 2010) defined by its unipolar cell lacunae. Semidentine cell lacunae were first identified in placoderms by Gross (1935) and were initially referred to as “Unipolare Knochenzellen” (unipolar bone cells), reclassified as the tissue “semidentine” by Ørvig (1951). Ørvig's reinterpretation was not initially accepted universally and the term was rejected by some authors, including Bystrow (1957) who preferred to interpret the tissue as bone, and Kulczycki (1957) who thought it transitional between bone and dentine. Enamel has not been found in the arthrodires; the thin layer of enamel identified in *Sedowichthys* by Bystrow (1957) has been widely disregarded, and Stensiö's (1925) enamel‐coated “coccosteid” jaw has been reinterpreted as acanthodian (Miles and Young, 1977). Bystrow (1957) identified an apparent evolutionary transition within arthrodires, from an ancestral state of thin plates bearing “dermal teeth” (with dentine and enamel) to an increasing thickness of plates and filling in of “teeth” with bone. Stensiö (1934) identified Haversian canals in the basal layer of *Phyllolepis*.

#### Antiarchi

Antiarchs also exhibit a three‐layered structure, with a superficial lamellar layer (termed the “Tuberkelschicht” by Gross, 1931), a cancellous spongiosa, and a compact basal lamellar layer (Grundlamellenschicht after Gross, 1931). This structure is typified by *Asterolepis ornata* (Heintz, 1929). Juvenile specimens of *A*. *ornata* and *Bothriolepis canadensis* exhibit three layers even in early ontogeny (Gross, 1931; Downs and Donoghue, 2009), with the compact layers being the first to develop. Antiarchs differ from Arthrodira in the presence of a basal cancellous component in the superficial layer. In addition, the superficial layer as a whole is separated from the middle spongiosa by a “planar discontinuity” (Goodrich, 1909; Gross, 1935). This was interpreted by Downs and Donoghue (2009) as a cementing line, and also as a plane of overlap between adjacent scarf joints. The superficial layer may possess tubercles that, in derived forms at least, are composed of cellular bone. It has been speculated that semidentine is present in ancestral antiarchs (Young, 2008), but this has not been substantiated. Both the superficial and middle layers contain evidence of extensive resorption and secondary bone growth (Downs and Donoghue, 2009). The presence of spheritic mineralization in all three layers of the dermoskeleton has led to a number of different interpretations. Stensiö (1931) considered the tissue to be an intermediary between bone and cartilage, whereas Ørvig (1968) classified it as “globular bone.” Burrow (2005) suggested the tissue was cartilage and thus part of the endoskeleton. Downs and Donoghue (2009), demonstrated that the topographic position of the spheritic mineralization, between dermal bone layers, is incompatible with an endoskeletal interpretation, instead interpreting the spheritic mineralization as evidence for rapid growth of dermal bone.

#### Ptyctodontida

**Figure 2:**
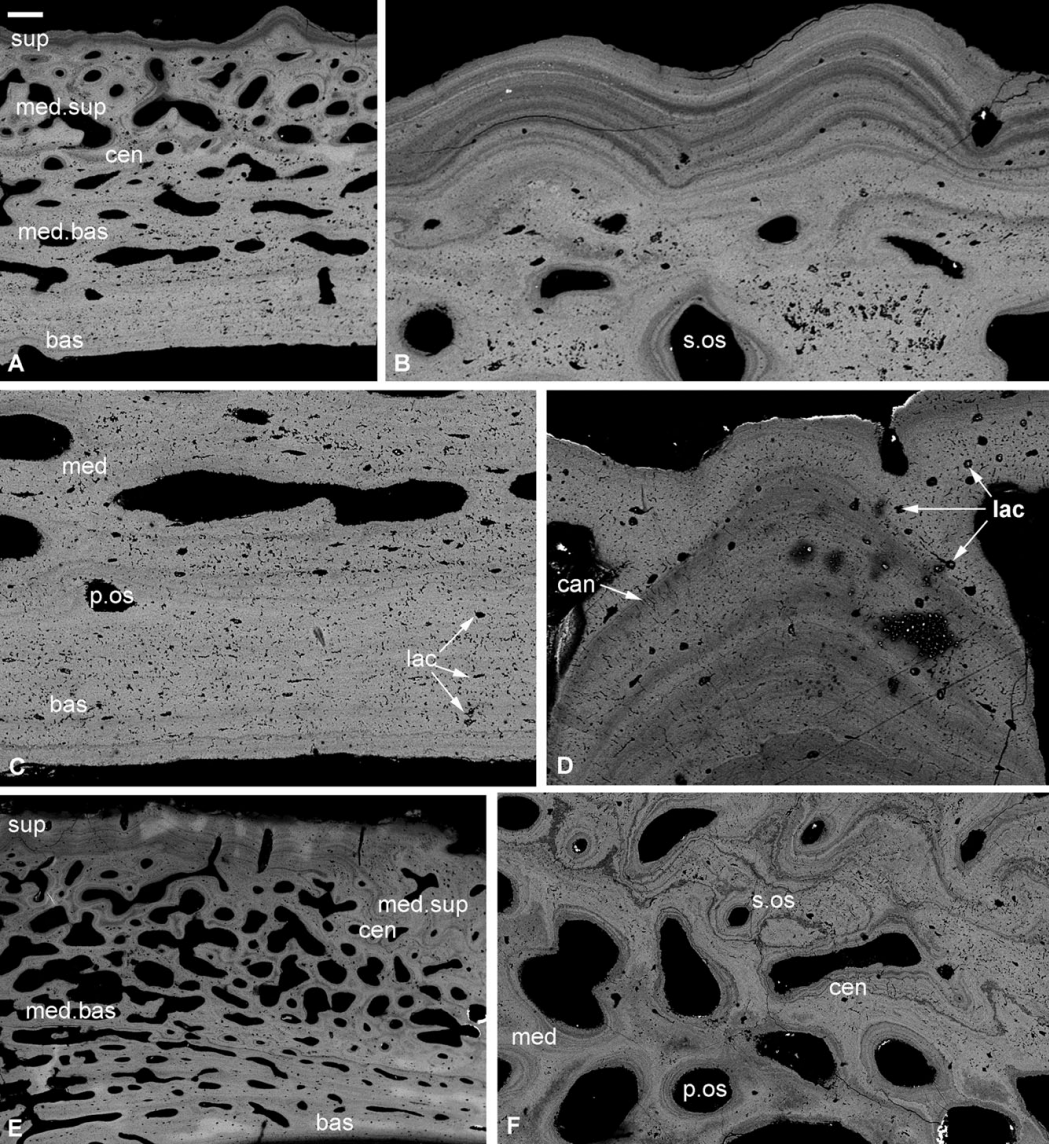
Histology of the dermal skeleton of the fossil arthrodiran placoderm *Incisoscutum* (**A**–**D**) and *Compagopiscis croucheri* (**E**, **F**). SEM images of NHMUK PV P 57639 and NHMUK PV P 50942. Section through three principal divisions (A), detail of the superficial layer and medial layer (B), detail of the medial layer and basal lamellar layer (C), and detail of the growth of tubercles (D). Three principal divisions at scarf‐joint with secondary osteons (E) and detail of secondary osteons (F). bas, basal lamellar layer; cen, central cancellous tissue; can, canaliculi; lac, lacunae; med, medial layer; med.bas, basal‐most zone of the medial layer; med.sup, superficial‐most zone of the medial layer; p.os, primary osteon; s.os, secondary osteon; sup, superficial layer. Scale bar equals 100 μm in (A), 40 μm in (B, C, F), 30 μm in (D), and 180 μm in (E).

The dermal skeleton has been interpreted as two‐layered, consisting of a compact surface layer and inner canal layer (Wells, 1944). Wells' figures contradict this simplistic interpretation, and a basal, possibly lamellar, layer appears to be present (Plate VII, Figs. 2 and 6, Wells, 1944). Compact dentine has been described in *Palaeomylus* (Wells, 1944), and acellular bone in *Palaeomylus* and *Eczematolepis* (Ørvig, 1980).

#### Acanthothoraci

Acanthothoracid histology is known only from scales of *Romundina* and *Murrindalaspis*. These contain an upper double‐layer of meso/semidentine, a middle spongiosa, and a lamellar base (Burrow and Turner, 1998). Tubercles might overgrow in three generations (Ørvig, 1975).

#### Petalichthyida

The histological description is incomplete. The surface layer in *Macropetalichthys* *agassizi* is made up of multiple generations of dentine tubercles (Gross, 1935), although this is not referred to by Wells (1944) in his investigation of *Macropetalichthys rapheidolabis*. In contrast, the tubercles of the primitive *Lunaspis* are made up of bone (Bystrow, 1957; at the time *Lunaspis* was considered an arthrodire). There is agreement that the spongiosa contains open and interconnected canals, but only Gross (1935) has figured a basal layer, which he noted is thin and often missing.

#### Rhenanida

Of the rhenanids, only the microstructure of *Ohioaspis tumulosa* scales has been investigated. Gross (1973) describes three layers of tissue: an “isopedinous” basal layer with possible evidence of bone reworking, a middle layer of vascular canals and previous generations of tubercles, and a “Skulpturschicht” of orthodentine‐like semidentine.

## MATERIALS AND METHODS

### Institutional/Geographic Abbreviations

ANSP, The Academy of Natural Sciences, Pennsylvania. BRSUG, Bristol University Geology Department. MNHN, Muséum national d'Histoire naturelle, Paris. NHMUK, The Natural History Museum, London. NSW, New South Wales, Australia. NRM, Naturhistoriska Riksmuseet, Stockholm.

### Materials

Thin sections are from the collection of the NHMUK: **Arthrodira**. *Taemasosteus* sp. median dorsal plate, Couvinian, NSW, Australia (NHMUK PV P.33620); *Holonema westolli*, Gogo Shales, Late Devonian, Australia (NHMUK PV P.67726). Specimens examined using scanning electron microscopy (SEM) and synchrotron radiation X‐ray tomographic microscopy (SRXTM) are stored in the ANSP, BRSUG, MNHN, NHMUK, and NRM: **Antiarchi**. *Chuchinolepis dongmoensis* posterior ventrolateral plate MNHN HISTPAL 2800 and *Yunnanolepis* sp. lateral plate, both from the Early Devonian, Lochkovian‐Pragian, Bac Bun Formation, Vietnam MNHN HISTPAL 2801. **Arthrodira**. *Antineosteus*, Emsian, Early Devonian, Tafilalt basin, eastern Anti‐Atlas, Morocco (BRSUG 29371.1); *Dunkleosteus* sp. suborbital plate, Famennian, Late Devonian, Mader basin, eastern Anti‐Atlas, Morocco (BRSUG 29371.2); *Compagopiscis croucheri*, Gogo, Western Australia (NHMUK OR 28E37, NHMUK PV P.50942); *Incisoscutum*, Late Devonian, Gogo Shales, Australia (NHMUK PV P.57639). **Ptyctodontida**. *Ctenurella* nuchal plate (NHMUK PV P.61507); *Ctenurella gardineri*, Late Devonian, Gogo Shales, Australia (NHMUK PV P.57665). **Phyllolepida**. *Phyllolepis* right anterior ventrolateral plate, Late Devonian, late Fammenian, Catskill Formation, Pennsylvania, United States of America (ANSP 21405). **Acanthothoraci**. *Romundina stellina* body scale (NRM‐PZ P.15952), triangular scale (NRM‐PZ P.15950), postorbital plate (NRM‐PZ P.15953), and unidentified skull plate (NRM‐PZ P.15951) from the Early Devonian, Lochkovian, Prince of Wales Island, Canada. **Petalichthyida**. *Lunaspis* anterior lateral plate, Devonian, Murrumbidgee Series, NSW, Australia (NHMUK PV P.50928).

### Scanning Electron Microscopy (SEM)

Specimens were set in Buehler EPO‐THIN® low viscosity Resin and sectioned using a Buehler IsoMet® low speed saw with a diamond saw blade. Where possible the sections were cut through natural margins and either parallel or perpendicular to the direction of growth. After sectioning, cut surfaces were impregnated with Buehler EPO‐THIN® within a vacuum chamber, to prevent loose particles coming free. The surfaces were ground briefly on 1200 μm silicon carbide paper and polished on mats using Buehler IsoCut® Fluid for lubrication. To remove scratches, Buehler MetaDi® 6 and 1 μm Diamond Paste was used. The samples were carbon coated using an Emitech K450 carbon coater and imaged using a Hitachi S‐3500N Scanning Electron Microscope. Backscattered electrons were used to image the tissues. Elements with a high atomic number appear brighter than those with a low atomic number as they backscatter more electrons, thus compositional changes within the tissue will show up as different brightness. This work was carried out at the University of Bristol School of Earth Science's Electron Microbeam Facility.

### Synchrotron Radiation X‐ray Tomographic Microscopy (SRXTM)

SRXTM was used at the TOMCAT beamline of the Swiss Light Source, Paul‐Scherrer Institut, Switzerland. SRXTM experiments followed a standard acquisition approach with the rotation axis located in the middle of the field of view and the acquisition of 1501 projections equiangularly distributed over 180°. Specimens were scanned using a 10× objective (resulting voxel size = 0.74 μm) and an energy of 21.5 keV (Fig. 7A–C).

### Light Microscopy (LM)

Thin sections were examined using at the University of Bristol using a Leica M250C microscope with a 2×Planapo lens and imaged with a Leica DFC425C digital camera.

## RESULTS

### Arthrodira

#### Incisoscutum

The outer surfaces of dermal cranial plates are densely covered with rounded, often connected tubercles, with small pores visible on the superficial surface. The dermal skeleton exhibits three principal divisions: a superficial lamellar tissue, a central cancellar tissue, and a basal lamellar tissue (Fig. 2A). *Superficial layer:* The superficial layer is compact and has a well organized matrix, with fibers arranged into multiple lamellae <1–20 μm thick. These lamellae have different atomic numbers, seen as different brightnesses (Fig. 2A,B,D), and as such represent lines of arrested growth, commonly interpreted as evidence of seasonal growth (de Ricqlès et al., 1991). They can either be parallel to the superficial surface—forming the external tubercles—or create the shape of internal tubercles (Fig. 2D). Cell lacunae are typically rounded and connected by multiple canaliculi, although they may occasionally be flattened and emit a single process parallel to the lamellae (Fig. 2B,D). There is no trace of semidentine within the tubercles, unlike those figured in *Incisoscutum ritchiei* by Johanson and Smith (2005; Text‐fig. 16F). Vascular canals are rare and approximately circular in cross section. While many of the canals are primary osteons, there is some evidence of remodeling by secondary osteons (Fig. 2B). These are distinguishable from primary osteons by the distortion of the preexisting fabric and the presence of a resorption line representing a spatiotemporal discontinuity. In primary osteons, the concentric lamellae forming the structure are deposited while bone growth is still active. In secondary osteons, the lamellae are deposited after bone growth has stopped, requiring some of the preexisting bone to be eroded away first. The superficial and medial layers are separated by a clear resting line in the form of less electron dense lamella (i.e., a layer with a higher atomic number, Fig. 2A). *Medial layer:* The middle layer is cancellar, with a less organized matrix than that seen in the superficial layer. There are frequent randomly oriented rounded to star‐shaped cell spaces, and a fairly high frequency of canaliculi. Canals have a rounded to elongate cross section and are often interconnected. Where elongate, the canals tend to be horizontal (relative to the basal and superficial surfaces) and extensive. There may be two zones of tissue (Fig. 2A): the superficial‐most zone contains larger, branching, irregular canals, which are typically surrounded by several concentric lamellae; the canals in the basal‐most zone are smaller and often elongate, not as interconnected as those above, and separated by much thicker trabeculae (Fig. 2A). The majority of vascular canals in the medial layer are primary osteons, although the prevalence of secondary osteons is higher in the superficial zone (Fig. 2A,C). The boundary between the cancellar and basal layers is much less distinct than that between the cancellar and superficial. The two tissues grade into each other, with the transition only occasionally marked by a change in electron density (Fig. 2A). The boundary is marked mainly by the disappearance of the canals seen in the medial tissue. *Basal layer:* The basal layer is compact with a faint lamellar matrix. The lamellae making up the layer are typically thicker than those in the superficial layer and are fewer in number (Fig. 2C). The cell lacunae display a considerable range of morphologies, from flattened and elongate to rounded or slightly star‐shaped (Fig. 2C). Canaliculi are dominantly parallel to the basal surface. The few canals that are present are roughly perpendicular to the lamellae, and occasionally open out on the basal surface. The interactions between the canals and lamellae imply a same‐age relationship, as in the superficial layer, with the external‐most lamellae having formed first.

#### Compagopiscis

Cranial dermal plates have rounded tubercles on their superficial surface, except on scarf joints (one section was oriented so as to intersect this scarf joint). The microstructure is similar to that in *Incisoscutum*, with a compact lamellar superficial layer, a cancellar medial layer, and a compact lamellar basal layer (Fig. 2E). *Superficial layer:* This tissue layer is compact, with the matrix organized into lamellae. The cell spaces in the superficial layer are circular in vertical section, although they can occasionally be flattened, and are connected by a large number of canaliculi. Vascular canals are largely absent. The layer is very thin or absent at scarf joints where canals of the medial layer (see below) extend to the surface. The superficial layer is separated from the underlying cancellar tissue by a thin resting line. *Medial layer:* Cancellar, with cell lacunae that are irregular in shape, from rounded to star‐shaped. Canaliculi are densely distributed. As in *Incisoscutum*, the cancellar layer is divided into two zones, with the upper zone pinching out beneath the scarf joint; secondary osteons are only found within the superficial‐most zone of this layer (Fig. 2F). The boundary, between the medial and basal layers, is often indistinct. *Basal layer:* Lamellae are present but poorly developed. Cell lacunae are rounded with a tendency to be flattened, and there are far fewer canaliculi than in either the medial or central layer. The layer also contains canals for Sharpey's fibers.

#### Holonema westolli

Dermal plates display three layers of tissue, similar to those seen in other arthrodires. *Superficial layer*: A thin cancellar layer with the matrix organized into faint lamellae. The superficial layer forms tubercles, which exhibit evidence of superimpositional stacking. No evidence of dentine is visible within the tubercles. Where visible, cell lacunae are rounded and connected by few canaliculi. The boundary between this and the medial layer is not obvious, with the two grading into each other. *Medial layer*: The medial layer is thick, making up most of the thickness of the plate. It is cancellar, with highly interconnected canals, and evidence for reworking in the form of secondary osteons is common. The matrix displays poorer organization than the matrix of the superficial layer. The boundary with the basal layer is marked by the appearance of lamellae. *Basal layer*: A fairly thin layer organized into lamellae. Occasional canals are present, although there is no evidence of reworking.

#### Antineosteus

**Figure 3:**
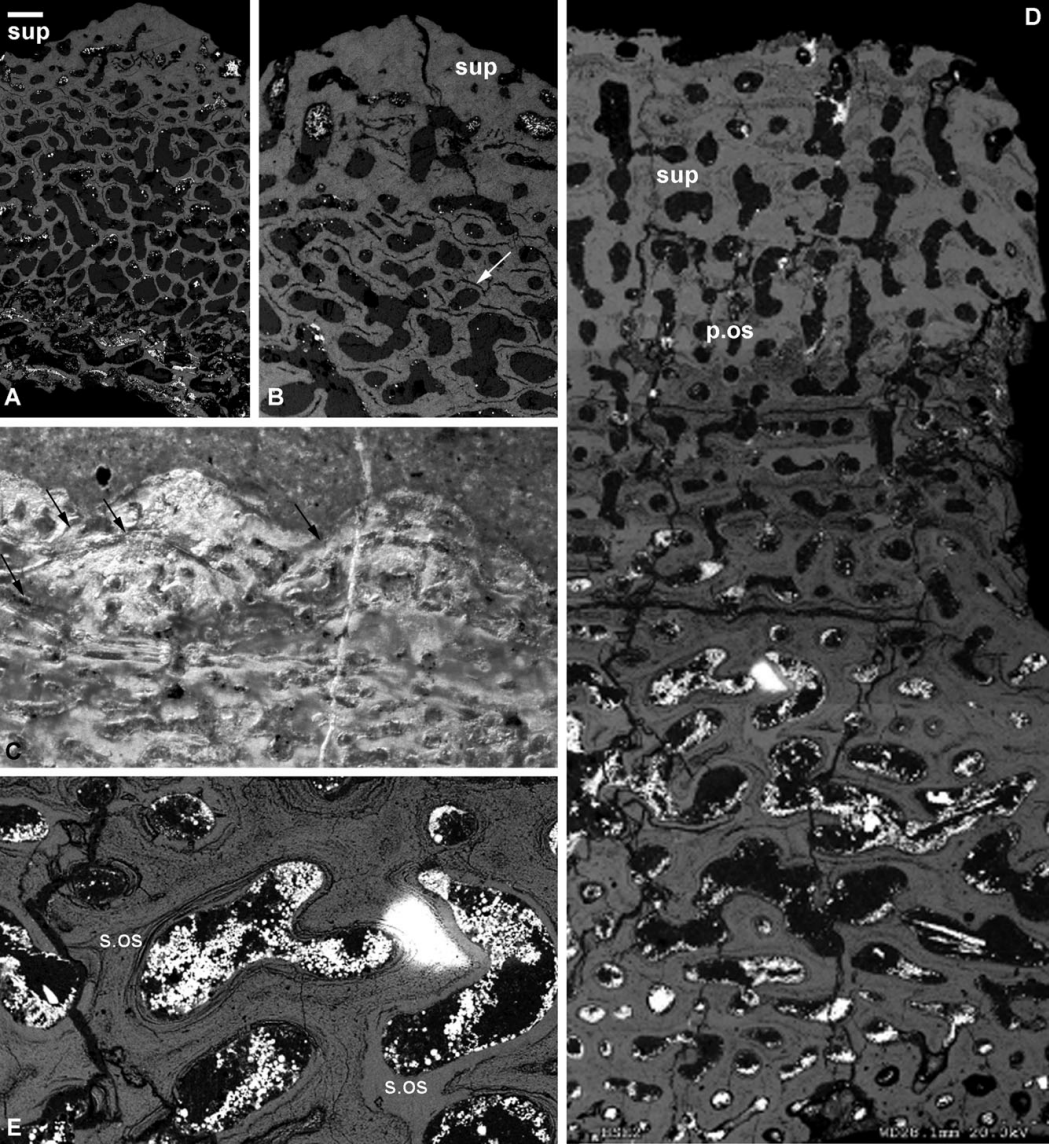
Histology of the dermal skeleton in the fossil arthrodiran placoderms *Antineosteus* (**A**, **B**) *Taemosteus* sp. (**C**) and *Dunkleosteus terrelli* (**D**, **E**). SEM images of BRSUG 29371.1 and BRSUG 29371.2 and LM images of NHMUK PV P.33620. Two principal divisions (A) and detail of the superficial and basal layer with cracks running midway between canals probably reflecting cementing lines (B). Appositional growth of superficial layer with truncated and overgrown tubercles (C), principal divisions of a superficial cancellar lamellar layer and a basal cancellar layer (D) and remodelling of the basal cancellous tissue (E). bas, basal lamellar layer; p.os, primary osteon; s.os, secondary osteon; sup, superficial layer. Scale bar equals 300 μm in (A), 150 μm in (B), 30 μm in (C), 200 μm in (D), and 100 μm in (E).

The dermal skeleton comprises two principal divisions: a thin, compact, alamellar superficial layer, seen only in the tubercles, and an open cancellar layer that extends to the basal surface (Fig. 3A). *Superficial layer:* Recrystallization has obscured the microstructure of the tissue matrix. The tissue is relatively thin (around 400 μm) and is only seen in the uppermost parts of the tubercles (Fig. 3A,B). No cell spaces and few vascular canals could be discerned due to diagenetic recrystallization of the mineral matrix. *Medial layer:* As with the superficial layer, it is not possible to distinguish the microstructure of the matrix. While there are no lamellae visible anywhere in the section, cracks running midway between canals may reflect cementing lines (cf. de Ricqlès et al., 1991; Fig. 5F; Fig. 3B). Cell lacunae do not appear to be present. The layer is made up of interconnected and branching canals, many of which have a subrounded cross section, separated by 5–15 μm thick trabeculae. These canals are often open on the basal and superficial layers. *Basal layer*: not observed.

#### Taemasosteus

The dermal skeleton comprises two principal divisions: a thin superficial layer and basally a thick cancellar layer. *Superficial layer*: The superficial layer appears confined to the tubercles. Tubercles are found both on the external surface of the superficial layer and internally. Many of these are truncated, and some can be completely covered by later growth (Fig. 3C). Rounded to elongate canals may be found within the tubercles, but there is no evidence of dentinous tissue. *Medial layer*: The medial layer accounts for almost the entire thickness of the plate. Cancellar and open with a disorganized matrix. In vertical section, two tissue “zones” can sometimes be discerned: an upper zone of rounded, irregularly connected canals, and a lower zone of larger, more elongate canals. This more basal zone extends to the base of the plate, and canals open onto the basal surface. *Basal layer*: not observed.

#### Dunkleosteus

Two distinct divisions of the dermal skeleton can be discerned: a superficial cancellar lamellar layer and a basal cancellar layer (Fig. 3D). *Superficial layer:* Cancellar and formed from bands of tissue with an organized lamellar matrix separated by bands of a more poorly organized tissue (Fig. 3D). Lamellae are 200–300 μm thick. Cell lacunae and canaliculi could not be discerned. Vascular canals within this layer are dominantly perpendicular to the superficial surface and are interspersed with some smaller canals. The vertical canals are evidence of rapid growth (de Boef and Larsson, 2007) and, as they cut through the lamellae, may provide evidence of an increase in the speed of growth late in ontogeny. Primary and secondary osteons are found within the superficial layer (Fig. 3D,E). *Medial layer:* The tissue making up the basal layer is poorly organized. Cell spaces are rounded, although few are present, and there are no obvious canaliculi. Canals with a circular cross section dominate the tissue immediately deep to the superficial layer and the basal‐most part of the plate. The intermediate area is filled with wider, more irregular interconnected canals. The level of reworking in *Dunkleosteus* is extensive. Many of the canals are surrounded by several concentric lamellae, and the lamellae, and the canals themselves, crosscut each other throughout the layer (Fig. 3E). *Basal layer*: not observed.

#### Phyllolepis

**Figure 4:**
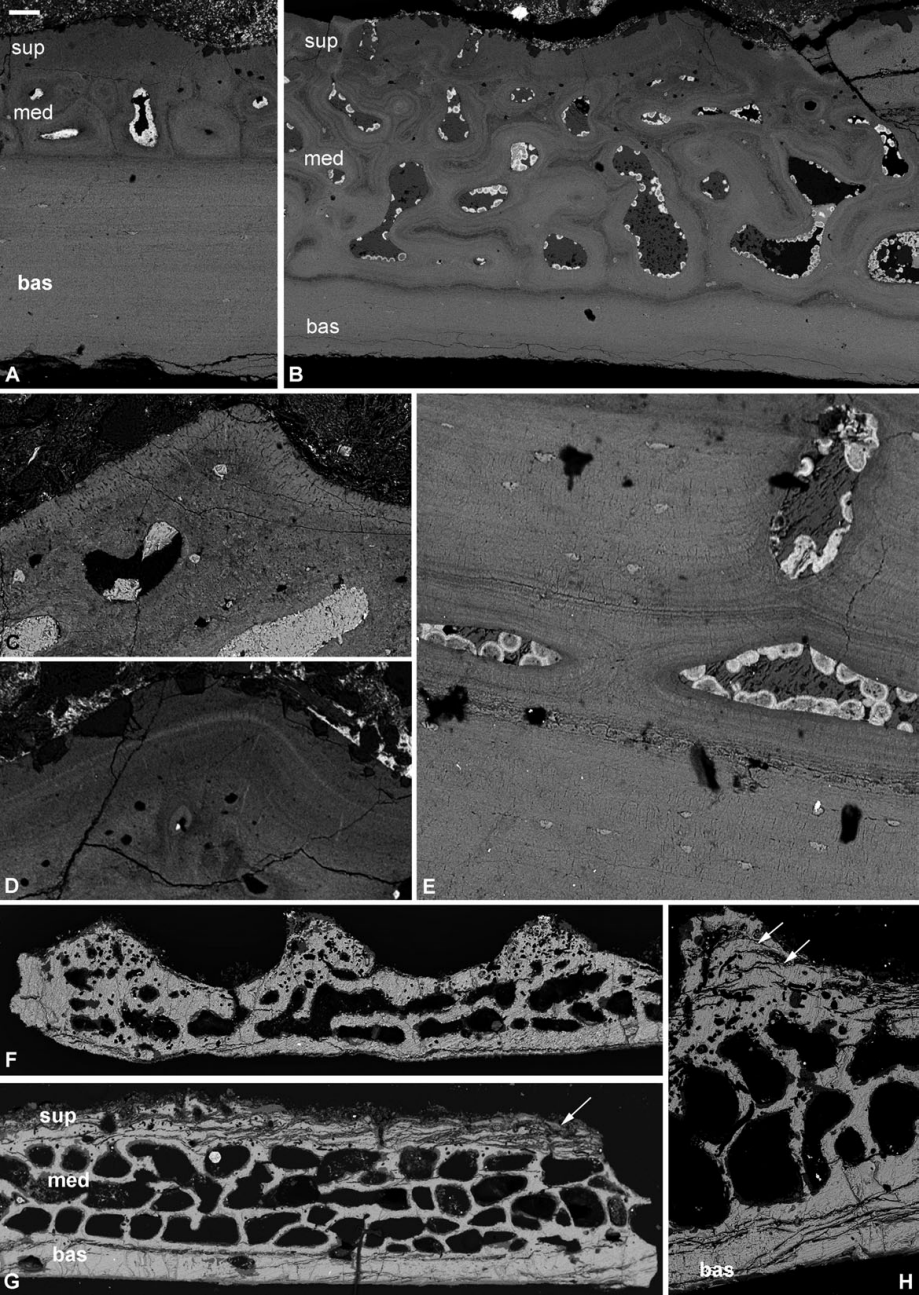
Histology of the phyllolepid arthrodire *Phyllolepis* (**A–E**) and the petalichthyid *Lunaspis* (**F–H**). *Phyllolepis* anterior lateral plate NHMUK PV P.50928: three‐layered structure with large basal layer (A), and large medial layer with cancellar spaces centripetally infilled (B), detail of the superficial layer with capping enameloid layer (C) and buried asymptotically tapering enameloid layer (D), section through a scarf joint basal layer with Sharpey's fibers and cell lacunae (E). *Lunaspis* ventrolateral plate ANSP 21405: three‐layered structure in peripheral, thinner part of the plate (F), three‐layered structure in thicker part of the plate with larger medial layer and parallel cracks in the superficial layer probably reflecting lamellae (G), detail of a tubercle showing cracks reflecting lamellae (H). bas, basal lamellar layer; en, enameloid; med, medial layer; sup, superficial layer. Scale bar equals 50 μm in (A), 67 μm in (B), 26 μm in (C), 30 μm in (D), 20 μm in (E), 157 μm in (F), 150 μm in (G), and 75 μm in (H).

The dermal skeleton comprises three principal divisions: a superficial lamellar layer, including the ornament; a medial layer with a large number of vascular canals; and a largely compact lamellar basal layer (Fig. 4A,B). *Superficial layer:* Compact, with fibers arranged to form faint lamellae approximately parallel to the superficial surface. The lamellae form gently undulating waves on the superficial surface. Cell lacunae are typically rounded but may be elongated perpendicular to the surface; canaliculi are abundant. Canals are almost entirely absent from this tissue. A small number of tubercles are capped by a thin (20 μm) electron‐dense layer (Fig. 4C,D), enveloping a matrix permeated by narrow dentine tubules that often bifurcate basally, and extend into the lamellar tissue deep to the surface. The matrix of the tissue comprising the bulk of the superficial layer is a less dense lamellar bone including round cell lacunae. The boundary between the superficial and medial layers is indistinct, with the two tissues intergrading. *Medial layer:* Cancellar, with much of the large cancellar spaces infilled with centripetally deposited lamellar bone including few rounded cell lacunae (Fig. 4A,B). The interstitial tissue comprising the initial walls between the cancellar spaces is too thin to resolve fine structure. The mottled appearance and rounded cell spaces within the tissue suggest it is woven/parallel fibered bone. The canals typically have a rounded or slightly elongate cross section, and in many cases have been surrounded by concentric bone lamellae. Canals near the terminal edges of the plate are surrounded by fewer lamellae. Although primary osteons occur densely within the medial layer. there is no evidence of resorption (contra Stensiö, 1934). There is a sharp difference in density between the medial and basal layers. *Basal layer:* The basal tissue is compact, lamellar, and encloses flattened cell lacunae with frequent canaliculi (Fig. 4A,B,E). Canals running roughly perpendicular to the basal surface represent channels for Sharpey's fibers. In sections that intersect a scarf joint, the basal layer incorporates a cancellar component in the middle of the layer (Fig. 4E). The canals are only one‐deep and are surrounded by concentric lamellae that do not distort or interfere with the lamellae of the basal layer. As cancellar bone is deposited at a faster rate than lamellar bone, the function of this may have been to allow the rate of growth at the joint to match that of the adjoining posterior median ventral plate. A similar argument was proposed by Downs and Donoghue (2009) to explain the function of the cementing layer described in *Bothriolepis canadensis*. The basal surface is normally planar, but towards the outer edges of the plate it undulates to produce low ridges that represent growth lines, although no corresponding growth lines (which would be perpendicular to the superficial and basal surfaces when cut) are preserved in the plate's microstructure. The basal layer varies greatly in thickness, from 150 μm near the edge of plates to nearly 400 μm in the center (Fig. 14F; the opposite of that observed by Stensiö, 1934, although this is likely to be an artifact of the position of the center of ossification). The thickest part of this layer corresponds with the center of ossification, with the medial layer being the thinnest.

#### Summary

**Figure 5:**
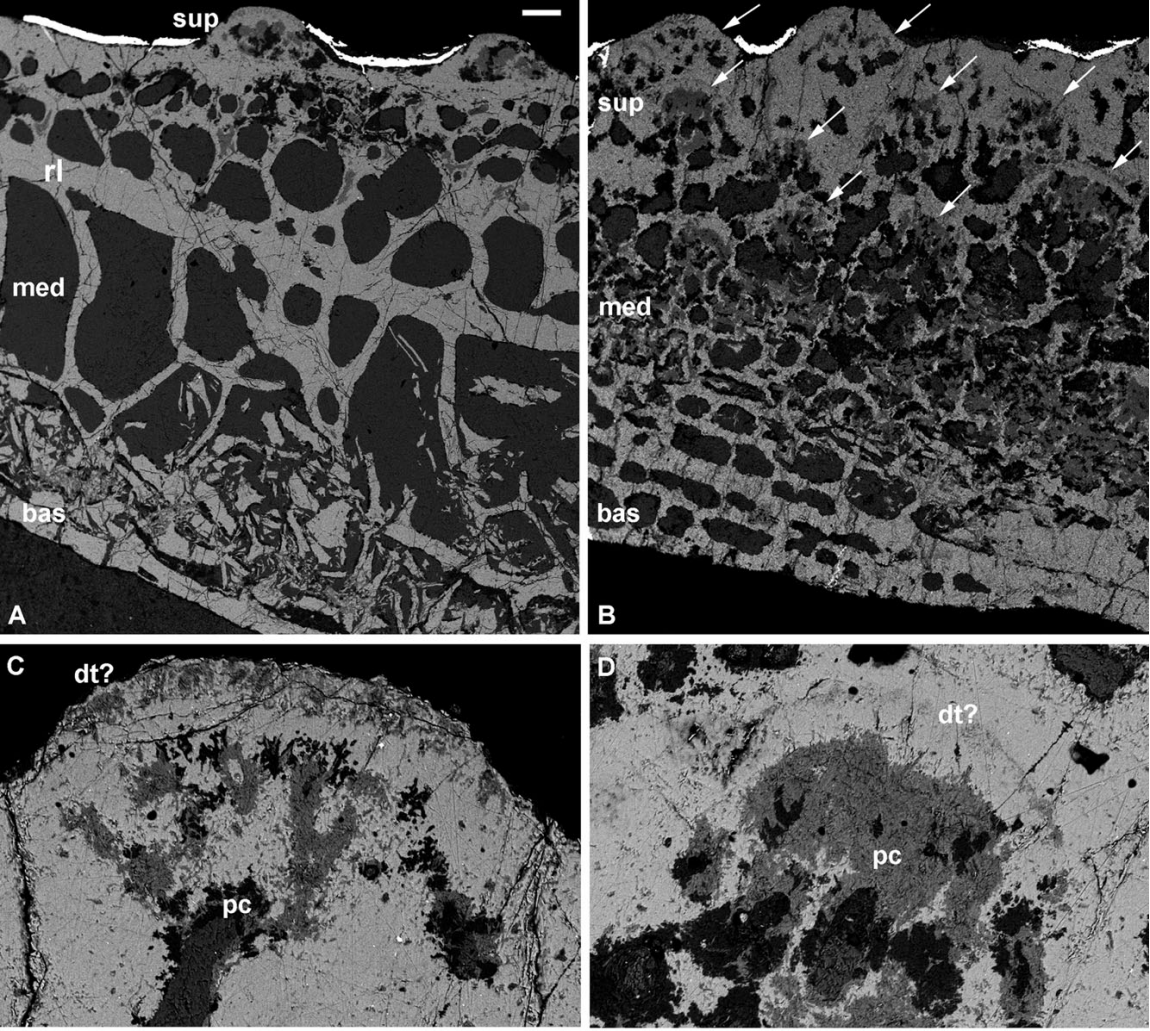
Histology of the basal fossil antiarch placoderm *Yunnanolepis* sp. SEM image of the lateral plate (MNHN HISTPAL 2801). Section through the three principal divisions (**A**), vertically stacked tubercles indicating appositional growth of the superficial layer, with arrows indicating the superficial surface of tubercles (**B**), tubercle in detail (**C**), and detail of overgrown tubercle (**D**). bas, basal lamellar layer; dt?, dentine; med, medial layer; pc, pulp cavity; rl, resting line; sup, superficial layer. Scale bar equals 158 μm in (A), 146 μm in (B), 27 μm in (C), and 29 μm in (D).

All of the arthrodires investigated are united in possessing cellular lamellar dermal bone overlying a division of cancellar spongy bone (with the exception of *Antineosteus*, which is extensively recrystallized). Although we found no evidence of dentine in the dermal skeleton, it has been reported in *Phlyctaenaspis* (Pl. II Fig. 5, Heintz, 1933) and certain buchanosteids (Text‐fig 3, Burrow and Turner, 1998). In *Phlyctaenaspis*, cell spaces have an elongate unipolar process, characteristic of semidentine (although Heintz describes them as unipolar bone cells). Remodeling of bone, in the form of surface overgrowth and resorption, is widespread in the arthrodires. These processes are well evidenced in *Coccosteus* and *Heterosteus* (Bystrow, 1957), as well as in buchanosteids (Text‐fig 3, Burrow and Turner, 1998). Marginal growth is seen in both *Incisoscutum* and *Compagopiscis*. At scarf joints, the medial cancellar layer is clearly differentiated into two zones, the upper zone of which exhibits greater evidence of remodelling than the lower.

### Petalichthyida

#### Lunaspis

The dermal skeleton exhibits a clear three‐layered structure: a superficial compact layer, which includes the external ornament, a medial cancellar layer, and a basal compact layer (Fig. 4F–H). *Superficial layer:* Although the tissue is clearly compact, fine detail of the matrix cannot be resolved due to diagenetic recrystallization. A number of cracks parallel to the superficial surface are present in this layer (Fig. 4G,H) and these likely betray an original lamellar structure. It is unclear whether the rounded holes in the tissue represent cell lacunae or postmortem damage. There are few canals but those present have circular cross sections. Although no dentine is present within these superficial ridges, dentine has been recorded comprising the tubercles of *Macropetalichthys agassizi* (Pl. II Fig. 5 and Text‐Fig. 9E, Gross, 1935) which exhibit evidence of vertical stacking from superpositional growth (Text‐Fig. 9C, Gross, 1935). As a consequence of recrystallization, the superficial layer appears to intergrade with the medial layer, with the boundary marked only by the appearance of larger vascular canals. *Medial layer:* The tissue contains canals with a rounded to rectangular cross‐section, which are only rarely interconnected. In thinner parts of the plate, these canals are only one deep (Fig. 4F), but in thicker parts they may be stacked three‐deep (Fig. 4G). Canals often truncate one another reflecting multiple episodes of growth. Again, the boundary between the medial and basal layer is indistinct, with the matrix of the basal tissue unclear. *Basal layer:* The tissue is compact and, as in the superficial tissue, cracks, possibly exploiting lamellae, run parallel to the external surface (Fig. 4H). Very few canals are present.

The nature of preservation means that the tissue matrix in the petalichthyids cannot be identified with certainty. Comparisons with other placoderm groups indicate that the tissues are likely to be composed of cellular bone.

### Antiarchi

#### Yunnanolepis

Three principal divisions comprise the dermal skeleton: a superficial layer with both compact and cancellar tissues, a medial cancellar layer, and a thin compact basal layer (Fig. 5A,B). The biological structure of the matrix and the nature of any cell lacunae are obscured throughout the plate due to recrystallization, giving the tissues a featureless, acellular appearance. *Superficial layer:* The superficial layer comprises an upper compact and a basal cancellar component. The compact tissue forms tubercles on the superficial surface (Fig. 5A–D). These are semicircular in shape and 100–200 μm in height. The fine detail of tissue structure is obscured by recrystallization, but the superficial layer is clearly composed of vertically stacked generations of tubercles with divided dentine pulp canals (Fig. 5C,D). The superficial and medial layers are separated by an 80–180 μm thick layer of a compact tissue (Fig. 5A), which is sometimes obscured due to disruption from canals or recrystallization. The position of this layer within the center of the plate suggests it is a cementing line, with the lack of resorption indicating a resting line. At 80–180 μm thick, this is too thick to represent a single resting layer, but may represent a series of them. *Medial layer:* The medial layer is cancellar, containing canals with a rectangular to flask‐shaped cross section separated by approximately parallel trabeculae. Near the basal surface, the trabeculae are often broken up and splintered by postmortem compaction (Fig. 5A,B). The boundary between the basal and medial layers is not identifiable by a change in tissue appearance. Rather, the basal layer is delimited by the extent of the canals in the central layer. *Basal layer:* The basal layer is formed from a 100 μm thick compact tissue (Fig. 5A,B). The layer is avascular.

#### Chuchinolepis

**Figure 6:**
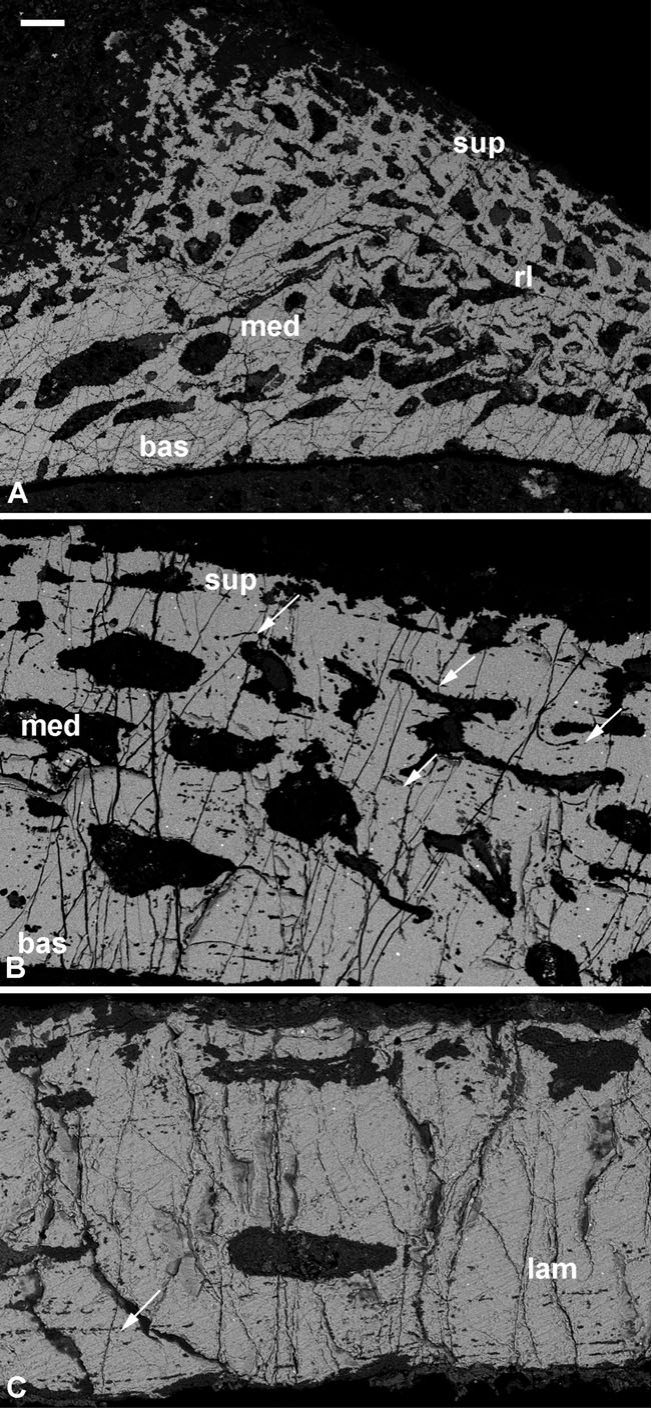
Histology of the basal fossil antiarch placoderm *Chuchinolepis dongmoensis*. SEM image of posterior ventrolateral plate (MNHN HISTPAL 2800). Three‐layered structure (**A**), middle layer with flattened lacunae (**B**), and basal layer with lamellae (**C**). bas, basal lamellar layer; med, medial layer; rl, resting line; sup, superficial layer. Scale bar equals 200 μm in (A), 60 μm in (B), and 50 μm in (C).

The microstructure of the dermoskeleton occurs in three principal divisions: a superficial layer with a thin upper compact and deeper cancellar component, a medial cancellar layer, and a basal compact layer (Fig. 6A). *Superficial layer:* The tissues have been recrystallized to such an extent that the fine detail of the matrix and cell spaces is obscured. The compact component is thin, with much of the layer represented by a cancellar component containing narrow, irregularly shaped canals. A 100 μm thick layer of compact tissue separates the superficial layer from the medial cancellar layer (Fig. 6A), although the extensive vasculature of the layers above and below results in this tissue often being obscured. *Medial layer:* Within the cancellar medial layer, the fine detail of the matrix is obscured and no cell spaces are visible. The canals are irregularly shaped, and many have been distorted during diagenesis. Many of the canals are surrounded by one or more concentric laminae, formed by minute flattened lacunae (Fig. 6B), which may be relics of original lamellae. There is no distinct boundary between the medial and basal layers. *Basal layer:* Compact, with sparse canals. Although no cell spaces are visible, laminae formed from lacunae form lines parallel to the basal surface (Fig. 6C). These may be exploiting preexisting lamellae.

#### Summary

Interpretation of the dermal skeletal tissues in *Yunnanolepis* and *Chuchinolepis* is difficult due to recrystallization. Comparison to published reports, however, shows that the three‐layered structure with a boundary of compact tissue between the superficial and medial layer is common to the antiarchs (Heintz, 1929; Downs and Donoghue, 2009). This allows the tissues to be interpreted in the light of published data. Downs and Donoghue (2009) found that the basal layer and the compact part of the superficial layer are lamellar and possess flattened to star‐shaped cell lacunae, leading to an interpretation of lamellar bone. The cancellar components are found to have a matrix of woven fibered bone. Extensive secondary osteons are also reported in all three layers (Text‐Figs. 4 A,D,F, Downs and Donoghue, 2009). It is probable that these features are also found in *Yunnanolepis* and *Chuchinolepis*. The pulp canals found within the tubercles of *Yunnanolepis* demonstrate the presence of dentine within the antiarchs for the first time.

### Ptyctodontida

#### Ctenurella sp

**Figure 7:**
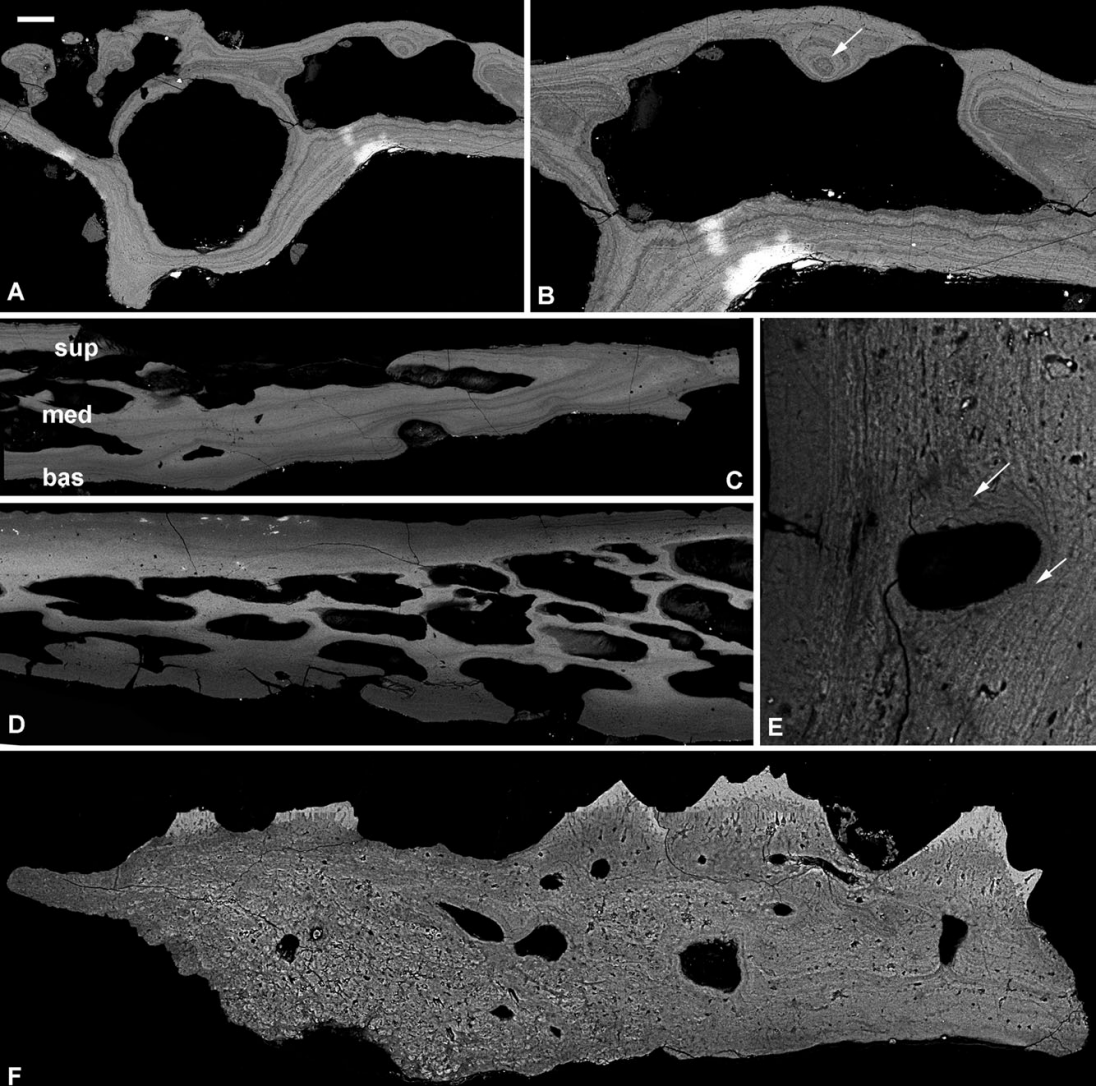
Histology of an early ontogenetic stage of the ptyctodont placoderm *Ctenurella* sp. (**A**, **B**), later stage of *Ctenurella gardineri* (**C**–**E**), and the acanthothoracid placoderm *Romundina stellina* (**F**). SEM images of probable nuchal plate NHMUK PV P.61507, a skull plate NHMUK PV P.57665 and a triangular scale NRM‐PZ P.15950. Lamellar bone layers (A), detail of centripetal addition of lamellae infilling vascular space (B), three‐layered structure at the periphery of the plate (C) three‐layered structure in the central of the plate (D), detail of external layer with multiple lamellae cross‐cutting external lamellae (E). Three‐layered structure of a triangular scale (F). bas, basal lamellar layer; med, medial layer; sup, superficial layer. Scale bar equals 60 μm in (A), 35 μm in (B), 100 μm in (C, D), 10 μm in (E), and 50 μm in (F).

Our material, from a juvenile, is very thin (390 μm at its thickest point). A hollow ridge, probably for housing a blood vessel, runs across the plate (Fig. 7A). The matrix of the plate is organized into lamellae that are a couple of microns in thickness. Rounded to slightly flattened cell lacunae are scattered apparently randomly through the plate. There is some evidence of centripetal addition of lamellae infilling the vascular spaces (Fig. 7B).

#### Ctenurella gardineri

The structure of the dermal skeleton is three‐layered, with superficial and basal compact lamellar tissues that join at the extremities of the plate, encapsulating a medial cancellar layer (Fig. 7C,D). *Superficial layer:* An ordered lamellar matrix and rounded cell spaces, which may emit a process. Canaliculi are sparse. Lamellae are typically parallel or subparallel to the superficial surface and up to 40 μm thick. Thinner lamellae may wrap concentrically around canals, and canals frequently interrupt the lamellae and open on the superficial surface. The superficial layer grades into the medial layer; there is no distinct boundary. *Medial layer:* The matrix of the cancellar layer is less well organized than that of the superficial layer. It has rounded cell spaces and a larger number of canaliculi. The canals are irregular and separated by thin trabeculae (∼20–50 μm thick). Canals can be up to three deep in the central, thickest parts of the plate (Fig. 7D), but at the periphery of the plate canals are only one deep. Again, there is no distinct boundary between the medial and basal layers, with one simply grading into the other. *Basal layer:* Much the same as the superficial layer, exhibiting a parallel fibered matrix with rounded to flattened cell lacunae. The basal lamellae fold down to form a triangular ridge, with a medial cancellar layer. Canals within the tissue may be surrounded by multiple lamellae that crosscut the lamellae of the external layer, providing evidence of remodelling, albeit on a small scale (Fig. 7E).

#### Summary

In both species of *Ctenurella* the dermal skeleton is composed of cellular lamellar bone. The medial cancellar layer in *C. gardineri* has a matrix is parallel fibered, although there are remarkably few canaliculi. As the younger specimen is formed only of lamellar bone, it may be surmised that the earliest bone laid down had a lamellar matrix surrounding a few large vascular spaces.

### Acanthothoraci

#### Romundina

**Figure 8:**
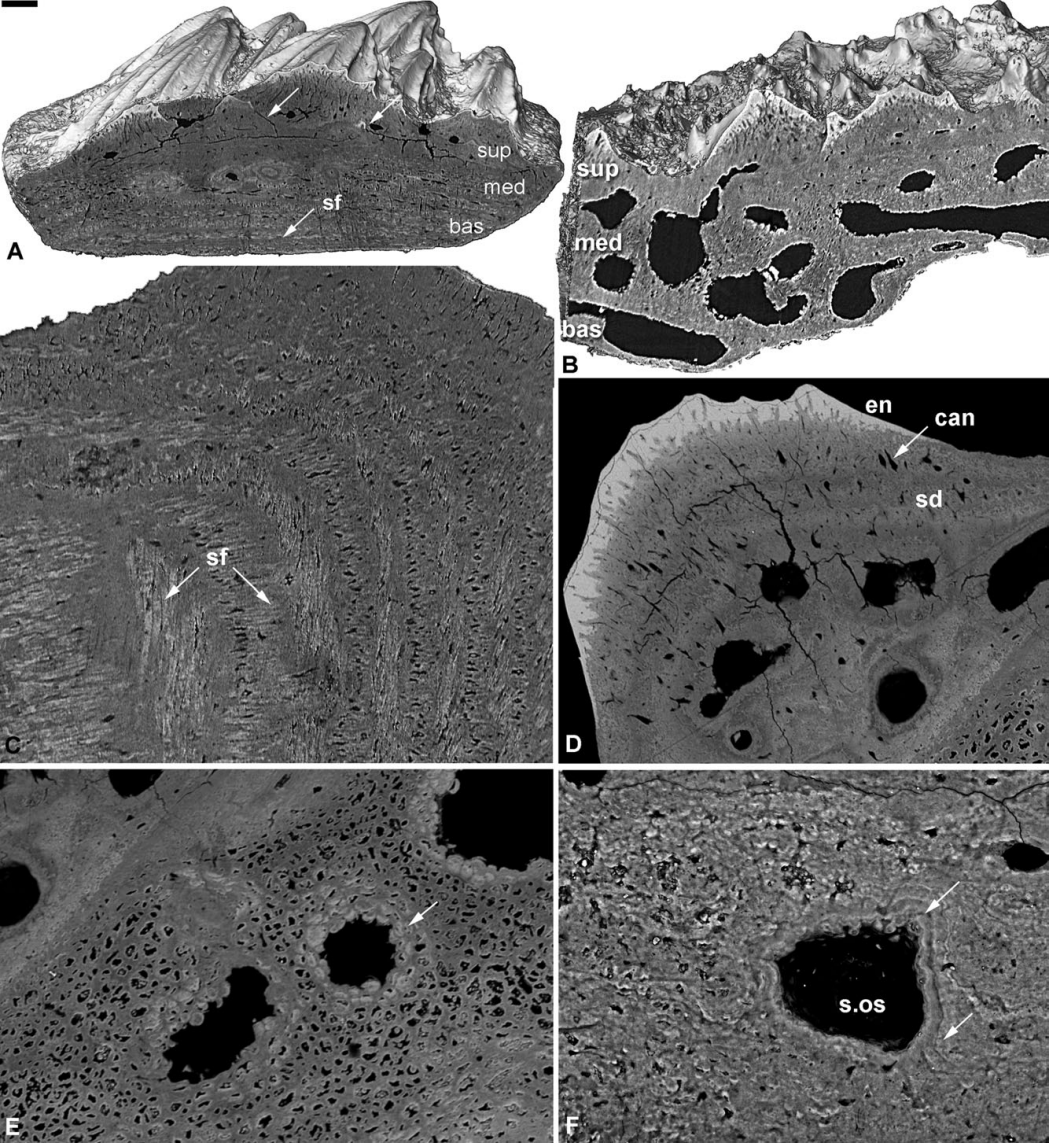
Histology of the acanthothoracid placoderm *Romundina stellina*. SEM images of a skull plate NRM‐PZ.15951, triangular scale NRM‐PZ.15950 and SRXTM images of postorbital plate NRM‐PZ.15953 and scale NRM‐PZ.15952. Three‐layered section of dermal scale with multiple generations of stacked tubercles (**A**), three‐layered structure of the cranial dermal skeleton (**B**), detail of the Sharpey's fibers in the basal layer (**C**), detail of the superficial layer with enameloid cap and centripetally layered semidentine forming a tubercle (**D**), detail of the spheritic mineralization within the medial layer (**E**), and detail of infill of a vascular canal (**F**). bas, basal lamellar layer; can, canaliculi; en, enameloid; med, medial layer; sd, semidentine; sf, Sharpey's fibers; s.os secondary osteon; sup, superficial layer. Scale bar equals 56 μm in (A), 55 μm in (B), 40 μm in (C), 15 μm in (D), 25 μm in (E), and 20 μm in (F).

The structure of the dermal skeleton is organized in three principle divisions: a superficial layer comprising tubercles, a cancellar medial layer, and a compact basal lamellar layer (Figs. 7F, 8A,B). *Superficial layer:* Composed of multiple generations of tubercles stacked vertically, with the largest and last‐formed tubercles on the upper surface of the dermal skeleton (Figs. 7F; 8B). The tubercles comprise a compact hypermineralized single crystalline enameloid monolayer (Figs. 6F, 8A,B,D) capping a core of centripetally layered semidentine exhibiting characteristic odontocyte lacunae with unipolar canaliculae (Fig. 8B,D) (Ørvig, 1975), although when cut in certain orientations odontocytes can appear almost cylindrical. The enameloid‐semidentine boundary is irregular since the odontocyte canaliculi extend into the enameloid layer (Fig. 8A,B,D), characteristic of single crystalline enameloid (Gillis and Donoghue, 2007). The semidentine odontoblasts are organized about a divided pulp cavity that overlies a more sparsely cellular region of cellular dermal bone that is equally well vascularized by primary osteons. The bony bases of the tubercles bind to the substrate of the medial division of the dermal skeleton or, in later generations, a substrate of earlier formed tubercles. *Medial layer:* The layer beneath the superficial tubercles exhibits two styles of mineralization. Frequently, the upper region is composed of spheritic mineralization where component spherites are 4–16 μm in diameter and have an unmineralized core, or are otherwise infilled with smaller spherites. The spherites exhibit a homogeneous distribution except around vascular spaces where they exhibit an osteonal organization into centripetal layers (Fig. 8E). The spheritic tissue layer is not always present, perhaps reflecting a mode of mineralization rather than a distinct histological layer. Where it occurs, the spheritic layer overlies a tissue with a rich polarized matrix of fiber bundles, organized into successive layers with alternating polarity, permeated by large cancellae. Where the spheritic tissue does not occur, this matrix‐rich tissue comprises wholly the medial division. *Basal layer:* A thin compact lamellar bone layer with sparse cell lacunae (Figs. 7A, 8A–C) permeated by extrinsic fiber bundles (Sharpey's fibers) (Fig. 8B,C). This tissue has also been reported in *Romundina* (Dupret et al., 2010) and in *Murrindalaspis wallacei* (Burrow and Turner, 1998; Text‐Fig. 8B).

**Figure 9:**
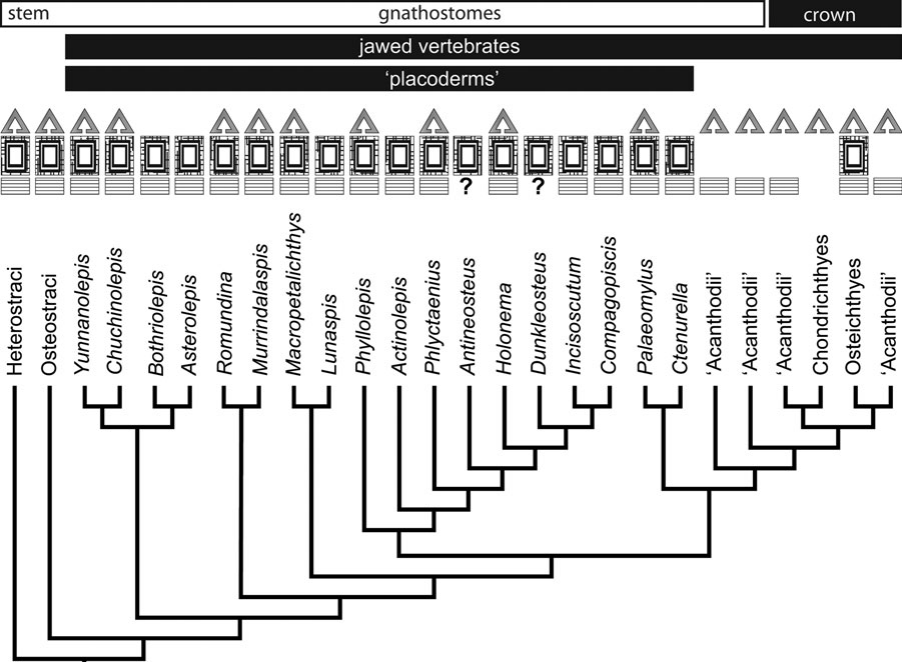
Phylogenetic relationships among the principal groups of placoderms and distribution of odontodes and tissue types. Phylogenetic relationships following the recent hypothesis of placoderm paraphyly (Johanson, 2002; Brazeau, 2009; Davis et al., 2012). Symbols represent the three‐layered structure of the dermal skeleton: triangles correspond to the superficial layer with tubercles containing enameloid or dentinous tissues; rectangles to the medial layer with cancellar bone; and line blocks to the basal layer with lamellar bone.

## DISCUSSION

### A Common Structure to the Dermal Skeleton in Placoderms

Our survey has revealed a greater diversity of dermal skeletal histology than has been considered hitherto. Nevertheless, this diversity appears to reflect a common structural organization although not every division is manifest in all taxa. The most complex structure is present in acanthothoracids, exemplified by *Romundina*, in which there are clearly three distinct divisions to the dermal skeleton, within which the superficial region is further differentiated into multiple generations of tubercles. Superficial tubercles are otherwise seen only in early antiarchs, petalichthyids, and early arthrodires; other placoderms exhibit a superficial layer of an appositionally accreted cellular tissue resembling dermal bone. Most placoderms exhibit a subdivided medial layer, but the medial layer is undivided in *Romundina* and some arthrodires. The medial division is usually composed of a spongy, cellular bone, sometimes exhibiting a fiber‐rich matrix. The medial division alone exhibits evidence of reorganization by resorption, through the action of secondary osteons, and their subsequent centripetal appositional mineralization. A basal division of appositionally accreted compact lamellar bone appears to be a general feature of the dermal skeleton in placoderms.

Deviations from this general organization are readily rationalized. In most placoderms that have been investigated, the medial division exhibits evidence of considerable reorganization through the ontogeny of the skeleton, first in the establishment of an upper and lower subdivision, and also through a secondary loss of this subdivision as a consequence of secondary osteonal activity. The absence of a medial subdivision in some taxa appears to reflect the initial subdivision not having occurred, rather than obliteration of its evidence by reorganization of the skeletal tissues.

Spheritic mineralization in the medial layer of *Romundina* occurs also in *Bothriolepis*, where it was originally interpreted as spheritic bone by Ørvig (1968). Burrow (2005) drew an obvious comparison between this style of mineralization and cartilage, concluding that the spheritically mineralized tissues in *Bothriolepis* represented part of the neurocranium, not the dermal skeleton. However, in *Bothriolepis*, as in *Romundina*, the spheritic mineralization is underlain by a distinct compact appositional lamellar bone layer that distinguishes the base of the dermal skeleton (Downs and Donoghue, 2009). Furthermore, in *Bothriolepis*, as in *Romundina*, the neurocranium can be distinguished as underlying the basal division of the dermal skeleton, unmineralized in *Bothriolepis* (Young, 1984), but perichondrally mineralized in *Romundina* (Ørvig, 1975). This style of mineralization is indicative of the absence of a coherent organic matrix (Ørvig, 1951), perhaps associated with rapid growth (Downs and Donoghue, 2009), and it does not appear to have phylogenetic significance.

### Evolution of the Dermal Skeleton in Early Jawed Vertebrates

The tripartite structure seen in placoderms is a general primitive feature for the vertebrate dermal skeleton, seen even in the earliest skeletonising vertebrates, such as the extinct jawless heterostracans (Donoghue and Sansom, 2002). It has been argued that a diagnostic character of placoderms is the presence of semidentine in the dermal skeleton (Denison, 1978; Goujet and Young, 2004; Young, 2008, 2010). However, it is clear from our survey that dentine of any kind is rarely present in the dermoskeleton of placoderms. It could be argued that semidentine is a shared primitive feature of the dermoskeleton of placoderms and, indeed, the presence of dentine is a shared primitive feature of the vertebrate dermal skeleton. Regardless, the presence of semidentine can hardly be considered a defining feature of placoderms if it is absent from the vast majority of placoderm taxa. Evidence for the secondary absence of tubercles from the superficial layer of the dermal skeleton in placoderms is supported by the presence of a capping monolayer of single crystallite enameloid associated with the tubercles in the acanthothoracid *Romundina*. This is an ancestral feature for the vertebrate dermoskeleton (Donoghue and Sansom, 2002; Donoghue et al., 2006) and, since acanthothoracids are held to be plesiomorphic placoderms regardless of whether placoderms constitute a clade (Young, 2010) or a grade (Brazeau, 2009; Davis et al., 2012), the absence of an enameloid capping layer associated with the tubercles in other placoderms suggests evidence of their progressive loss through phylogeny.

It has been argued that widespread bone remodelling of the dermal skeleton is restricted to sarcopterygians (Zhu et al., 2010). However, it has already been demonstrated that the antiarch *Bothriolepis* exhibits evidence of extensive remodelling of the dermal skeleton (Downs and Donoghue, 2009). We have shown that this is a widespread phenomenon of the cranial dermal skeleton in placoderms, achieved through the action of secondary osteons in the middle layer of not just antiarchs, but ptyctodonts, and arthrodires, too. We found no evidence of secondary osteons in phyllolepids (contra Stensiö, 1934). In gnathal elements, there is centripetal growth within primary vascular structures by dentinous tissues in arthrodires, initiated by the superficial layer, but there is no remodelling of the superficial layer of tubercles or teeth (Ørvig, 1980; Johanson and Smith, 2005; Rücklin et al., 2012). The argument presented by Zhu et al. (2010) concerns only the superficial layer of dermal tubercles and, in agreement with this view, we found no evidence of resorption in the superficial layer in placoderms.

Perhaps the key distinctions of the dermal skeleton in placoderms are the subdivision of the medial division and, perhaps ironically, the appositional superficial layer of cellular bone that occurs in placoderms that lacks dermal tubercles and their attendant semidentine. The subdivision of the medial layer may be associated with the development of the overlapping scarf joints between adjacent cranial dermal plates (Downs and Donoghue, 2009). Regardless, although neither character is encountered in stem‐gnathostomes (perhaps with the exception of galeaspids; Wang et al., 2005), the development of the superficial division through vertical apposition of cellular bone is a characteristic of the cranial dermal skeleton in stem‐tetrapods, many of which exhibit a stratified medial division (Witzmann, 2009; Zylberberg et al., 2010). Whether this similarity reflects convergence or common descent depends upon the condition of the cranial dermal skeleton in phylogenetic intermediates, which need not consider whether placoderms are monophyletic or paraphyletic. Unfortunately, there is a paucity of evidence on the histological structure of the cranial dermal skeleton at the base of the actinopterygian and sarcopterygian stem‐lineages because most histological research has focussed on the structure of the postcranial dermal skeleton.

Our survey of the placoderm skeleton, supplementing published knowledge, supports phylogenetic loss of dentine tubercles from the superficial division of the dermal skeleton in many of the component placoderm clades (Fig. 9). This is convenient since the implications for the evolution of the vertebrate dermal skeleton remain the same, regardless of which of the two prevailing phylogenetic hypotheses on placoderm relationships is the closest approximation of the truth. Given the traditional hypothesis of placoderm monophyly (Fig. 1b), the plesiomorphic condition for the placoderms is a three‐layered cranial dermal skeleton with a superficial layer of dentine tubercles capped with a monolayer of single crystallite enameloid, since this is a feature of acanthothoracids, which are perceived as the sister group to all other placoderms (Goujet and Young, 1995; Young, 2010), and successive sister groups to placoderms share these characteristics (Donoghue and Sansom, 2002; with the exception of the enigmatic galeaspids; Wang et al., 2005). The current prevailing hypothesis of placoderm paraphyly (Fig. 1a, 9) identifies antiarchs as the earliest jawed vertebrates, comprising the first of a series of sister lineages that is followed in sequence by acanthothoracids, arthrodires, or ptyctodonts, a subset of acanthodians and, ultimately, crown‐gnathostomes (Johanson, 2002; Brazeau, 2009; Davis et al., 2012). The principal difference in the evolutionary scenario implied by this phylogenetic framework (Fig. 9) is that the same grade of placoderm dermoskeleton was maintained for a longer phylogenetic interval by early jawed vertebrates. Under either scenario, dentine tubercles are lost within the plesia of the gnathostome stem group, rather than within the lineage itself. Hence neither scenario implies phylogenetic incongruence in the homology of the superficial dentine tubercles that are of course retained in chondrichthyans. Similarly, under either phylogenetic framework, there is evidence among jawed vertebrates within the gnathostome stem for a shift from large dermal plates comprising the cephalothorax, to the small polyodontode scales that comprise the cranial dermal skeleton and retain a medial and basal division in acanthodians (Hanke et al., 2001; Hanke and Wilson, 2004; Blais et al., 2011) and early chondrichthyans (Karatajuté‐Talimaa, 1992; Miller et al., 2003). Homologies have long been sought between the skull table of placoderms and osteichthyans (Graham‐Smith, 1978). Reconciling whether or not the skull table of osteichthyans is neomorphic, or a vestige of the stem‐gnathostome cranial dermal skeleton, will depend heavily upon resolving the histological structure of the cranial dermal skeleton in stem‐osteichthyans, knowledge of which is currently in disarray (Friedman and Brazeau, 2010).

## CONCLUSIONS

The dermal skeleton of placoderms exhibits the same tripartite structure seen in the earliest skeletonising vertebrates, with a superficial layer, medial layer, and basal layer being present. Dermal tubercles comprising enameloid and dentinous tissues occur in the acanthothoracids, inherited from jawless relatives, but are absent through loss in more derived placoderm lineages. Semidentine has been considered a synapomorphy of placoderms, yet our data show that while dentine occurs plesiomorphically as part of the dermal skeleton in each of the component clades of placoderms, it is absent from derived members of these lineages, although it remains associated with the gnathals as part of the splanchnocranium. Remodelling of the dermal skeleton through ontogeny occurs rarely in stem gnathostomes and it has been considered a synapomorphy of osteichthyans. We show that remodelling is widespread among placoderms, particularly antiarchs and arthrodires, but it appears restricted to the medial division of the dermal skeleton.
